# Dr. John E. Brady

**Published:** 1914-07

**Authors:** 


Dr. John E. Brady, Buffalo, 1860, died at his home in Grand Rapids, Mich., May 18, aged 77. He was Asst. Surgeon of the 45th Illinois Volunteers, during the Civil War; a teacher in the Grand Rapids Medical College; and coroner of Kent Co., Mich.

